# miR-130b, an onco-miRNA in bladder cancer, is directly regulated by NF-κB and sustains NF-κB activation by decreasing Cylindromatosis expression

**DOI:** 10.18632/oncotarget.10423

**Published:** 2016-07-06

**Authors:** Xiaolu Cui, Chuize Kong, Yuyan Zhu, Yu Zeng, Zhe Zhang, Xiankui Liu, Bo Zhan, Chiyuan Piao, Zhenming Jiang

**Affiliations:** ^1^ Department of Urology, The First Hospital of China Medical University, Shenyang 110001, Liaoning, China

**Keywords:** bladder transitional cell carcinoma, NF-κB, miR-130b, CYLD

## Abstract

Persistent activation of NF-κB signaling is closely related to chronic inflammation and tumorigenesis. Commonly, NF-κB signaling is tightly controlled by multiple feedback loops and regulators, such as the deubiquitinases (DUBs). However, in cancer cells, NF-κB may override these feedbacks through special pathways and lead to the sustained activation. In the present study, we demonstrate that in transitional cell carcinoma (TCC) of bladder, miR-130b plays an oncogenesis role, it enhanced proliferation, invasion and migration of TCC cell, and was highly correlated with tumor progression. On the other hand, NF-κB directly regulated the transcription of miR-130b by binding with its promoter region. Importantly, we verify that, through deceasing the expression of Cylindromatosis (CYLD), a K63-specific DUB and endogenous blocker of NF-κB signaling, miR-130b can in return sustain the persistent activation of NF-κB, which may promote the malignant progression of TCC. Thus, the present study uncovers a potential signaling transduction in which NF-κB is continuously activated, and may provide a novel therapeutic approach for the clinical management of TCC.

## INTRODUCTION

Bladder cancer is the second most commonly diagnosed genitourinary malignancy worldwide and is the most common malignancy in China [1,2]. Transitional cell (urothelial) carcinoma (TCC) accounts for approximately 95% of bladder cancers [3]. Although approximately 70% of newly diagnosed TCCs of the bladder are found to be non-muscle-invasive [1,2], which can present as a non-lethal disease initially, 47% of these tumors recur and 9% will eventually progress to a muscle-invasive bladder cancer [1,2], which is commonly associated with a high risk of death due to distant metastases [3]. Cisplatin-based chemotherapy has been shown to be efficient in controlling some muscle-invasive bladder cancers; however, it has less potential to improve survival in other bladder cancers [4]. Although mechanism-based targeted therapies have excelled in many other cancers in the last decade, such as sunitinib in kidney cancer and gefitinib in lung cancer, there is still no such targeted therapeutic reagent approved by the Food and Drug Administration to treat TCC of the bladder [1,2,4]. Thus, in attempts to develop a more efficient therapeutic strategy, studies on the mechanism underlying tumor development and progression in bladder cancer are urgently needed.

The nuclear factor-kappa B (NF-κB) family, which includes NF-κB1/p105/p50, NF-κB2/p100/p52, RelA/p65, RelB, and cRel, has been widely studied in the field of immunology and cancer biology [5,6]. Persistent activation of NF-κB can not only lead to chronic inflammation but can also result in tumorigenesis [7]. Bound by the inhibitor of NF-κB (IκB), NF-κB exists in its inactive state as homodimers or heterodimers in the cytoplasm [8], among which, the classical NF-κB P50/P65 heterodimer is the most abundant of the Rel/NF-κB dimmers and plays a more elaborate role than other factors in regulating gene expression [9]. In contrast, IκB kinase (IKK) complex is able to phosphorylate complex-associated IκB, resulting in the ubiquitination and degradation of IκB [10]. When released from blocking by the IκBs complex, NF-κB rapidly accumulates in the nucleus, where it binds to the DNA at κB sites, which are present within the promoters and enhancers of hundreds of genes and regulate gene expression [11]. Thus, ubiquitination plays a central role in the activation of NF-κB [7,10,12,13]]. In contrast, ubiquitination is counter-regulated by a family of deubiquitinases (DUBs) [7,13,14], which counteract E3 ubiquitin ligases and inactivate NF-κB signaling [15,16]. Cylindromatosis (CYLD), one of the DUBs, also known as a tumor suppressor gene, directly interacts with NF-κB essential modulator (NEMO), which is a regulatory subunit of NF-κB, and with tumor necrosis factor receptor (TNFR)-associated factor 2 (TRAF2); thus, it negatively regulates NF-κB activity [14,17,18].

MicroRNAs (miRNAs) are an abundant class of highly conserved endogenous small non-protein-coding RNAs. To the primary knowledge, miRNAs function as negative regulators of gene expression by base pairing with the 3′-untranslated region (3′-UTR) of their target mRNA, causing RNA degradation or translation suppression. There are also studies purposed that miRNAs are capable of activating gene expression in respond to certain cell types and conditions [19]. Accumulating evidence has shown that miRNAs play important roles in homeostatic processes, such as cell formation, cell proliferation, and cell death [20–22]. It has also been firmly established that the dysregulation of miRNAs is linked to the initiation and progression of human malignances when they are associated with tumor suppressors, oncogenes, or other genes involved in cell differentiation [23–25]. The microRNA-130 family has been reported to be linked to different types of cancers, including gastric cancer [26], colorectal cancer [27], pancreatic cancer [28], renal cell cancer [29], glioma [30], hepatocellular carcinoma [31], endometrial cancer [32], and papillary thyroid carcinoma [33]. There have also been studies showing that miR-130b could be a potential diagnostic and prognostic biomarker for bladder cancer [34,35] because the level of miR-130b is significantly increased in the sera of patients with bladder cancer. However, the functional role of miR-130b in TCC, as well as its regulatory mechanism, remains largely unknown.

In our study, we demonstrated that miR-130b was an onco-miRNA in TCC of the bladder because it was not only significantly upregulated in TCC cell lines and tissue samples, but it also promoted the proliferation, invasion, and migration of bladder cancer cells. Importantly, the expression level of miR-130b in muscle-invasive tissue samples was significantly higher than that in non-muscle-invasive tissue samples, indicating its potential association with poor prognosis in patients with bladder cancer. Furthermore, we identified that NF-κB directly regulated the transcription of miR-130b and that CYLD, an endogenous NF-κB-negative regulator, was a bona fide target of miR-130b. These findings suggested that, by upregulating the expression of miR-130b and consequently inhibiting CYLD, NF-κB sustained its persistent activation and stimulated the progression of bladder cancer. Therefore, the NF-κB/miR-130b/CYLD axis could promote the progression of bladder cancer and could provide potential targets for cancer therapy.

## RESULTS

### miR-130b was upregulated in TCC tissues and cell lines and was correlated with the progression of bladder cancer

**Figure 1:**
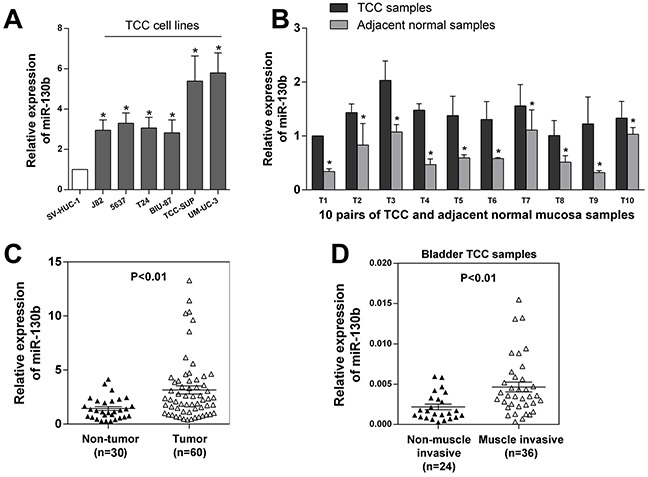
miR-130b expression was upregulated in TCC tissues and cell lines and was correlated with the progression of bladder cancer **A.** miR-130b expression levels in one normal urothelial cell line and six bladder cancer cell lines were analyzed by real-time PCR. **B.** miR-130b expression levels were detected in 10 pairs of TCC and adjacent normal mucosa tissue samples. **C.** miR-130b expression levels in 30 normal bladder mucosa tissue and 60 TCC tissue samples. These results indicated that miR-130b was significantly upregulated in TCC cell lines and tissues. **D.** miR-130b expression levels in 24 non-muscle-invasive and 36 muscle-invasive tissue samples. miR-130b expression was found to be remarkably upregulated in muscle-invasive tissues, suggesting that high miR-130b expression was very likely to be correlated with poor prognosis. The expression levels were normalized to U6. Each bar represents the mean ± SD of three independent experiments. *p < 0.05.

Using quantitative reverse transcription-polymerase chain reaction (qRT-PCR), we first assessed the expression of miR-130b in six TCC cell lines (5637, J82, BIU, T24, TCC-SUP, UM-CM-3), as well as in one normal urothelial cell line (SV-HUC-1). As shown in Figure 1A, the expression of miR-130b was found to be significantly elevated in all of the TCC cell lines compared with the normal cell line. Next, we assessed the expression of miR-130b in 10 freshly collected TCC samples and the corresponding adjacent normal bladder mucosa. Again, increased expression of miR-130b was observed in all of the tumor samples compared with their paired normal controls (Figure 1B). To confirm this finding, we further assessed the expression of miR-130b in 20 other archived non-tumor tissue samples and 50 other archived TCC tumor tissue samples. Indeed, the average level of miR-130b was found to be dramatically elevated in the tumor samples compared with the normal controls (Figure 1C).

Furthermore, we assessed the associations of the level of miR-130b expression with the clinical and pathological characteristics of these tumors. As shown in Table 1, miR-130b was more highly expressed in high-grade tumors than in low-grade tumors. Additionally, miR-130b was found to be upregulated more frequently with increasing tumor stage, suggesting a potential correlation of the expression of miR-130b with tumor progression. In contrast, there was no significant association found in the expression of miR-130b with regard to lymphatic invasion. This finding may have been due to the relatively small sample size because there were only 6 cases with lymphatic invasion among our samples. Importantly, when we measured and analyzed miR-130b expression in TCC tissue samples with different T stages, we found significant upregulation of miR-130b in the muscle-invasive group compared with the non-muscle-invasive group (Figure 1D). This finding revealed that miR-130b may be an independent prognostic factor for bladder cancer. Together, these findings suggested that the elevated expression of miR-130b was associated with the increase in tumor aggressiveness, commonly leading to poor prognoses of patients.

**Table 1:** Associations between miR-130b and clinicopathological characteristics

Parameters	Number of cases	miR-130b		*P* value
		High	Low	
**Sex**				
Male sex, N(%)	49(81.7)	34(85)	15(75)	0.345
**Age(y)**				
Age ≥65, N(%)	37(61.7)	26(65)	11(55)	0.575
**Histologic grade**				
High grade, N(%)	41(68.3)	31(77.5)	10(50)	0.011*
**T stage**				
>pT2, N(%)	15(25)	13(32.5)	2(10)	0.042*
>pT1, N(%)	36(60)	29(72.5)	7(35)	0.002**
**Lymphatic invasion**				
Positve, N(%)	6(10)	6(15)	0(0)	0.058
**Distant metastasis**				
Positive, N(%)	0			

The cut-off point was determined by youden index to reach the highest values of sensitivity and specificity for T stage classification (> pT2).

* Statistically significant (P < 0.05).

** Statistically significant (P < 0.01).

### miR-130b overexpression increased the proliferation of TCC cells in vitro and enhanced tumor growth in vivo

**Figure 2:**
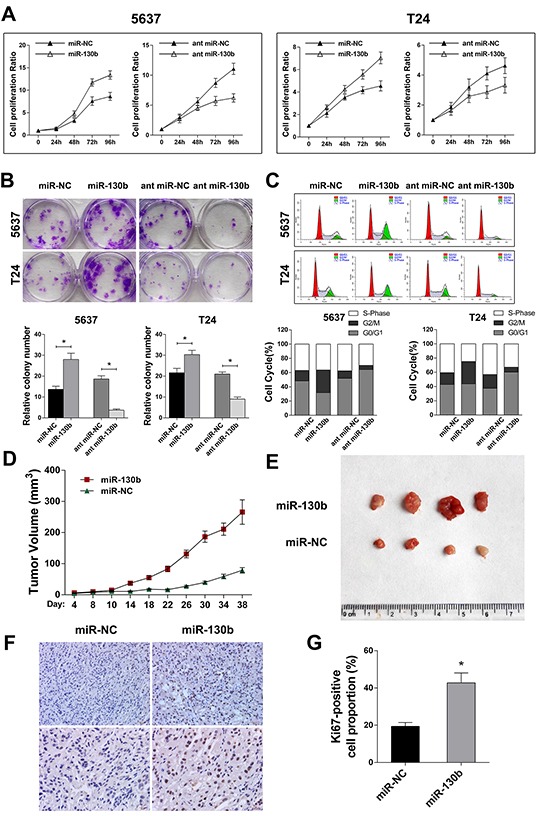
miR-130b overexpression increased the proliferation of TCC cells in vitro and enhanced tumor growth in vivo **A.** The cell proliferation was evaluated by CCK8 analysis on the indicated day after transfection. **B.** Crystal violet-stained colonies of 5637 and T24 cells were captured and counted. Overexpression of miR-130b significantly increased the proliferation rates of TCC cells, while inhibition of miR-130b expression suppressed cell proliferation. **C.** Flow cytometry cell cycle analysis of bladder cancer cells. Cells in G2/M phase were markedly increased in miR-130b overexpression group compared with the control, whereas cell cycle was significantly arrested in G0/G1 phase in miR-130b knockdown group. **D.** Xenograft model in nude mice (n = 4/group). Tumor volumes were measured on the indicated days and are presented as the mean ± SD. **E.** Images of the excised tumors from each group. **F, G.** Staining of Ki-67-positive cells from the tumors. Original magnification: 200X (upper); 400X (lower). The proliferation index was determined by counting the Ki-67-positive cells. Each bar represents the mean ± SD of three independent experiments. *p < 0.05.

To investigate the involvement of miR-130b in tumor progression, TCC cell lines 5637 and T24 were transfected with the agomir or antagomir of miR-130b, as well as their respective negative controls (NCs). The transfection efficacy was assured by qRT-PCR (Supplementary Figure S1A). After transfection, we performed CCK8 analysis and colony formation assays to evaluate the effects of miR-130b on cell proliferation. As shown in Figure 2A and 2B, overexpression of miR-130b significantly increased the proliferation rates of 5637 and T24 cells, while inhibition of miR-130b expression suppressed cell proliferation. Similarly, flow cytometry showed that cells in the G2/M phase were markedly increased in miR-130b overexpression group compared with the control, whereas cell cycle was significantly arrested in G0/G1 phase in miR-130b knockdown group (Figure 2C), suggesting a role of miR-130b in promoting cell proliferation.

To examine the effects of miR-130b on the progression of bladder cancer *in vivo,* nude mice were inoculated with 5637 cells. After the volume of the xenograft tumors reached approximately 3 mm^3^ (approximately 1 week after inoculation), the mice were randomly divided into two groups (n = 4/group). The agomir of miR-130b or negative control was injected intratumorally twice per week in both groups. The results showed that the tumors in the mice that received an agomir injection grew much faster than those in the mice that received the control treatment (Figure 2D, 2E). Further histological examination showed that treatment with miR-130b agomir increased the expression of Ki67 in xenograft tumor cells compared with the control treatment (Figure 2F, 2G). This result suggested that, in agreement with the proliferating effect of miR-130b on bladder cancer cells *in vitro*, overexpression of miR-130b enhanced tumor growth *in vivo*.

### miR-130b stimulated invasion and migration of TCC cells and induced epithelial mesenchymal transition (EMT) of cells

**Figure 3:**
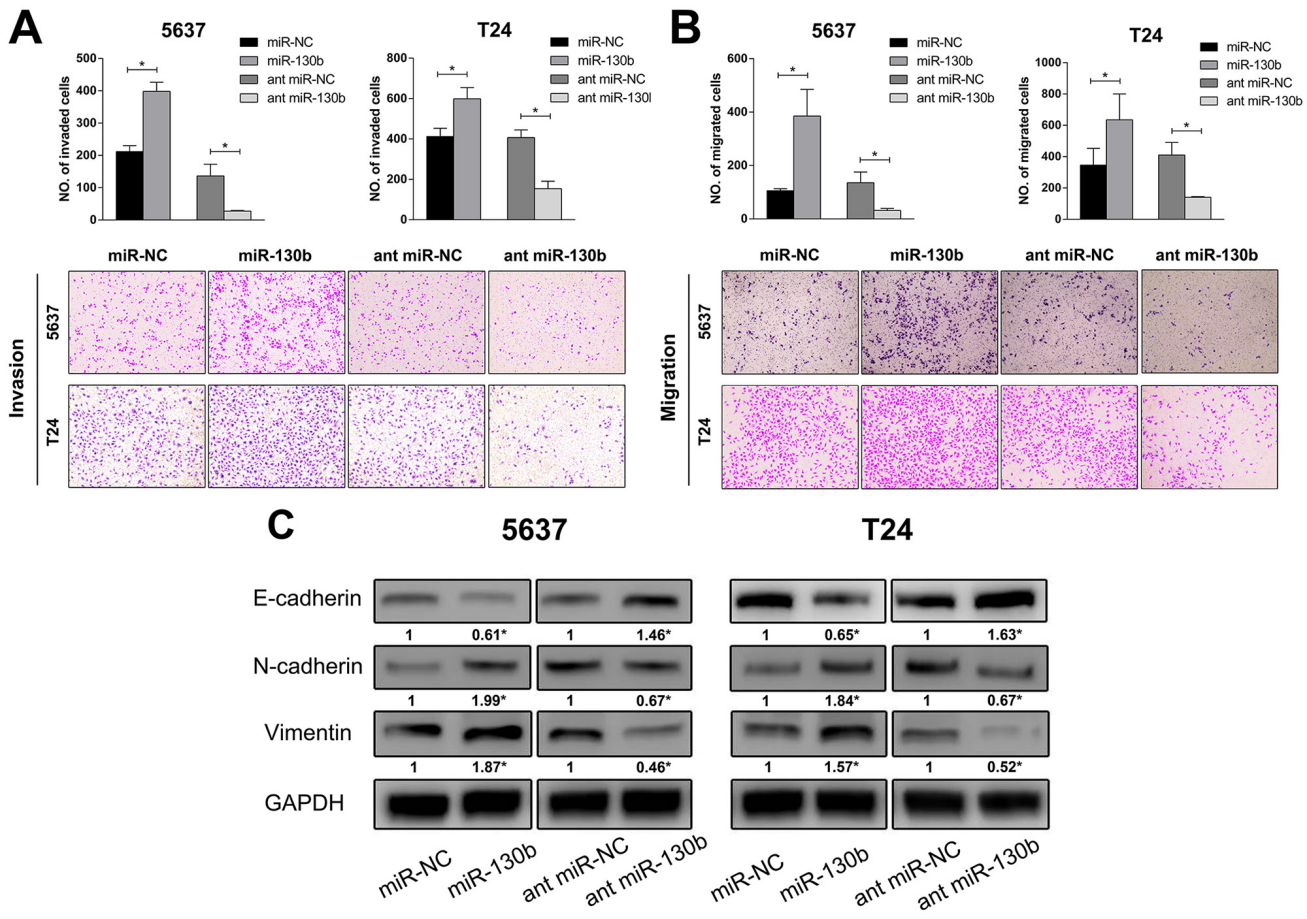
miR-130b stimulated the invasion and migration of TCC cells and induced epithelial mesenchymal transition (EMT) of cells **A, B.** Invasion and migration assay of bladder cancer cells. Cells were fixed and stained with crystal violet 48 hours after transfection, and images were captured (magnification: 200X). Overexpression of miR-130b stimulated the invasion and migration of 5637 and T24 cells. **C.** EMT-related proteins were analyzed by Western blotting. Overexpression of miR-130b increased the expression of N-cadherin and vimentin and decreased the expression of E-cadherin, while inhibition of miR-130b increased the expression of E-cadherin and decreased the expression of N-cadherin and vimentin. These results suggested that miR-130b induced epithelial mesenchymal transition of TCC cells, and was likely to stimulate cell invasion. The densitometric values were calculated using AlphaEaseTM FC software. GAPDH served as a loading control. The ratio of target protein to reference protein was used to conduct the statistical analysis. Each bar represents the mean ± SD of three independent experiments. *p < 0.05.

To further explore the effects of miR-130b on tumor progression, the invasion and migration abilities of bladder cancer cells were assessed by the transwell assay with either over-expression or inhibition of miR-130b in these cells. As shown in Figure 3A, for both the 5637 and T24 cell lines, the number of invaded cells was remarkably higher in the miR-130b overexpression group than in the control group, while the inhibition of miR-130b impaired the invasion of these cells through the membrane. The migration assay revealed similar results (Figure 3B). These data suggested that, in addition to increasing cell proliferation, miR-130b was also capable of enhancing cell invasion and migration of bladder cancer. In light of the mechanism underlying the effects of miR-130b on cancer cell invasion and migration, we aimed to determine whether miR-130b induced epithelial mesenchymal transition (EMT) in bladder cancer cells. To assess this possibility, the expression of three EMT markers (E-cadherin, N-cadherin, and vimentin) was examined by western blot analysis. Indeed, we observed that the overexpression of miR-130b inhibited the expression of E-cadherin and increased the expressions of N-cadherin and vimentin, whereas the inhibition of miR-130b expression reversed the expression patterns of these proteins (Figure 3C). These results indicated that miR-130b induced the EMT process and was likely to stimulate the cell invasion of bladder cancer.

### NF-κB induced miR-130b expression

**Figure 4:**
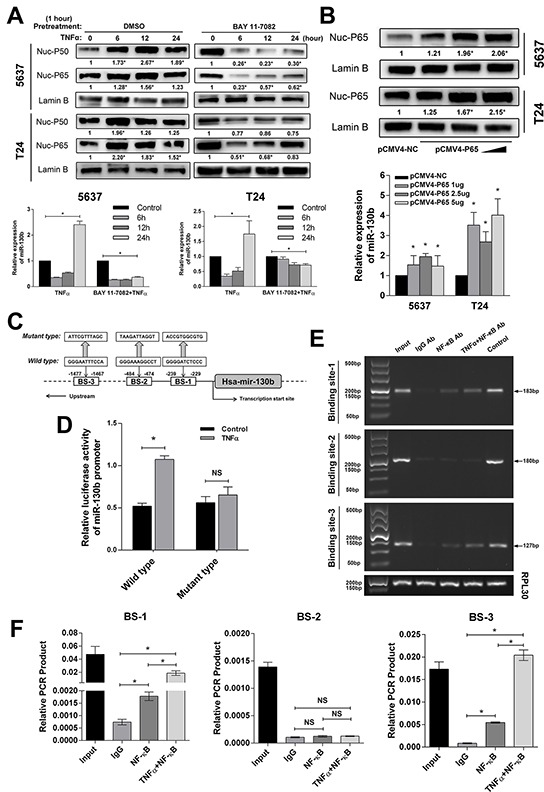
NF-κB directly bound with the promoter region of the miR-130b gene and induced miR-130b expression **A.** 5637 and T24 cells were treated with TNF-α (10 ng/ml) for 6, 12, and 24 hours. Nuclear proteins were extracted, and NF-κB subunits P50/P65 in nuclei were measured by western blot analysis. In another group, 5637 and T24 cells were pretreated with BAY 11-7082 for 1 hour and were then treated with TNF-α (10 ng/ml) for 6, 12, and 24 hours. NF-κB subunits P50 and P65 in nuclei were measured by western blot analysis. Lamin B served as a loading control. The ratio of target protein to reference protein was used to conduct the statistical analysis. The miR-130b level was analyzed by real-time PCR. The expression levels were normalized to U6. **B.** The tested cells were transfected with pCMV4-P65 plasmid in increasing concentrations. Nuclear P65 was detected by western blot analysis. miR-130b expression was analyzed by real-time PCR. Each bar represents the mean ± SD of three independent experiments. *p < 0.05. **C.** Schematic of a typical miR-130b promoter. The predicted NF-κB binding sites were located at −229 to −239, −474 to −484, and −1467 to −1477. Two hundred base pairs upstream of the miR-130b promoter region were cloned into a pmirGLO luciferase reporter plasmid. For the mutant type, nucleotides at three predicted binding sites were altered simultaneously. **D.** Also shown are luciferase assay results. Activation of NF-κB significantly increased the luciferase activity of the wild-type miR-130b promoter. 5637 cells were seeded in a 24-well plate and were separately transfected with wild/mutant types of miR-130b promoter for 24 hours. Then TNF-α (10 ng/ml) was added to the medium, whereas control wells were changed with serum-free medium. Cells were incubated for another 24 hours, and luciferase and renilla signals were measured by microplate reader. **E.** Chromatin immunoprecipitation (ChIP) assay results showed that NF-κB physically bound with the miR-130b promoter. Precipitate DNA was amplified using specific miR-130b promoter primers for 25 cycles and was resolved on 1% agarose gel. *Lane 1*, input chromatin prior to immunoprecipitation. *Lane 2*, immunoprecipitation with a non-specific antibody (IgG). *Lane 3*, immunoprecipitation with NF-κB P50 antibody. *Lane 4*, immunoprecipitation with NF-κB P50 antibody when NF-κB signaling was hyperactivated. *Lane 5*, immunoprecipitation with histone H3 antibody. **F.** qRT-PCR was also performed to measure the enrichment of predicted binding fragments. Each bar represents the mean ± SD of three independent experiments. *p < 0.05.

Given the likely oncogenetic function of miR-130b in bladder cancer, as shown above, we further investigated whether its regulation was involved in some important pathways related to cancer development or progression. First, we analyzed the promoter region of the miR-130b gene using 4 different types of software: PromoterScan (http://www-bimas.cit.nih.gov/molbio/proscan/), Jaspar (http://jaspar.genereg.net/), Promoter, version 2.0 (http://www.cbs.dtu.dk/services/Promoter/), and ChipBase (http://deepbase.sysu.edu.cn/chipbase/). Interestingly, all of the bioinformatics data indicated that there were NF-κB response elements within the miR-130b promoter. To verify this finding, 5637 and T24 cells were treated with 10 ng/ml tumor necrosis factor-α (TNF-α) to activate NF-κB signaling; then, miR-130b expression was examined after 6, 12, and 24 hours. To measure the activation of NF-κB, nuclear P50 and P65 proteins were assessed by western blot analysis (Figure 4A), while the mRNA expressions of three NF-κB downstream factors, namely, NFKB inhibitor alpha (NFKBIA), TNF alpha induced protein 3 (TNFALP3), and interleukin-8 (IL-8), were detected by qRT-PCR (Supplementary Figure S1B). As the results show in Figure 4A, in both the 5637 and T24 cells, miR-130b was significantly upregulated after 24 hours of TNF-α treatment. However, in contrast, we noted that, at 6 and 12 hours, miR-130b levels were decreased. Furthermore, the cells were pretreated with NF-κB inhibitor BAY 11-7082 (an selective inhibitor against IKK1 and IKK2, capable of blocking the phosphorylation of IκBα induced by TNF-α) for 1 hour; then, the cells were treated with TNF-α as described. As expected, we found that the activation of NF-κB was remarkably suppressed due to the blocking effect of BAY 11-7082 (Figure 4A). At the same time, pretreatment with BAY 11-7082 significantly inhibited TNF-α-induced miR-130b expression as early as 6 hours after treatment (Figure 4A). Considering the complex functions of TNF-α that may induce varies of pathways, which may result in the unexpected repression of miR-130b in our experiment, we next directly activated NF-κB by transfecting P65 plasmid with different concentrations of 1μg, 2.5μg and 5μg (Figure 4B, Supplementary Figure S1C). Again, miR-130b expression was found to be significantly increased by P65 transfection in a dose-dependent manner (Figure 4B). Together, these results demonstrated that the activation of NF-κB induced miR-130b expression, whereas the inhibition of NF-κB signaling suppressed miR-130b expression.

### NF-κB directly bound with the promoter region of the miR-130b gene

The three predicted potential NF-κB binding sites (BS) in the promoter region of miR-130b are shown in Figure 4C. To experimentally verify the direct binding of NF-κB at these sites, a pmirGLO luciferase reporter plasmid, containing an miR-130b promoter sequence with or without mutant bases, was constructed. Data from the dual luciferase reporter assay showed that TNF-α treatment significantly induced luciferase activity in the cells that were transfected with the construct containing wild-type miR-130b promoter but not in the mutant construct-transfected cells (Figure 4D). Furthermore, the chromatin immunoprecipitation (ChIP) assay revealed that NF-κB physically bound with the promoter region of the miR-130b gene (Figure 4E, 4F). These results strongly suggested that NF-κB induced miR-130b expression through direct enhancement of its transcription.

### miR-130b inhibited CYLD expression and activated NF-κB signaling

**Figure 5:**
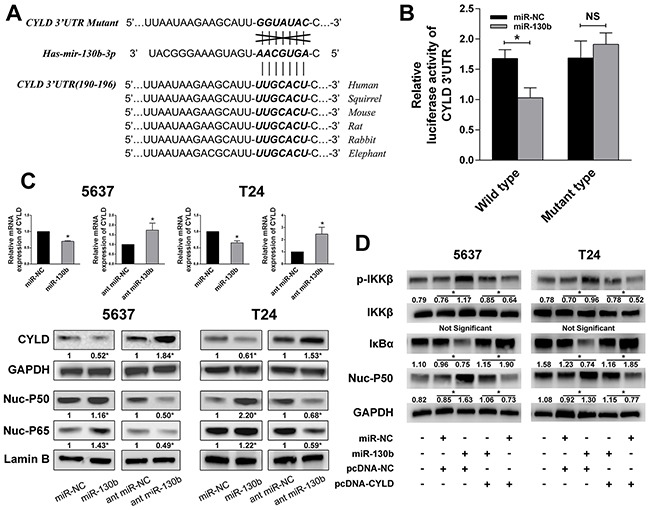
miR-130b inhibited CYLD expression and activated NF-κB signaling **A.** The highly conserved CYLD 3′UTR and predicted miR-130b target sequence in the 3′UTR of CYLD and the mutant type with 7 altered nucleotides. **B.** The luciferase assay was performed with cotransfection of miR-130b agomir and wild-type or mutant-type CYLD 3′UTR in 5637 cells. Firefly luciferase activity of each sample was normalized against renilla luciferase activity. **C.** Overexpression of miR-130b significantly decreased the mRNA and protein levels of CYLD. NF-κB subunits were also measured, and NF-κB activation was detected in miR-130b overexpression group. **D.** miR-130b overexpression induced the phosphorylation of IKK, decreased IκB expression and resulted in NF-κB activation. In contrast, miR-130b overexpression with CYLD restoration had no effect on IKK phosphorylation or IκB degradation. Moreover, CYLD overexpression led to the dephosphorylation of IKK, upregulated IκB expression and inhibition of NF-κB activity. All data from three separate experiments are presented as the means ± SDs. *p < 0.05.

Next, we explored the potential targets of miR-130b interaction using Targetscan (http://www.targetscan.org/vert_50/seedmatch.html) and found that CYLD, a crucial negative regulator of NF-κB, was a putative target of miR-130b (Figure 5A). The dual luciferase reporter assay was again performed to measure the binding of miR-130b with the 3′UTR region of CYLD mRNA. The pmirGLO luciferase reporter plasmid, containing a wild-type or mutant-type CYLD 3′UTR, was co-transfected with miR-130b agomir or scrambled control. As shown in Figure 5B, overexpression of miR-130b decreased the luciferase activity of the cells transfected with the construct containing the wild-type CYLD 3′UTR, whereas it had no effect when the cells were transfected with the construct bearing the mutant type. qRT-PCR and western blot analyses confirmed that miR-130b overexpression significantly decreased the mRNA and protein levels of CYLD in the tested cells (Figure 5C). Furthermore, we investigated whether miR-130b affected NF-κB activation by analyzing the translocation of NF-κB subunits to the nucleus, and we found that the overexpression of miR-130b resulted in increased NF-κB activation, whereas the downregulation of miR-130b decreased NF-κB activation (Figure 5C). To further validate the assumption that miR-130b activates NF-κB signaling by decreasing CYLD expression, the pcDNA-CYLD plasmid and the relative empty vector were employed. We co-transfected the cells with miR-130b/pcDNA-NC or miR-130b/pcDNA-CYLD, and we measured the phosphorylated IKK, IKK, IκB, and nuclear NF-κB subunits by western blot analysis. Consistent with our expectations, miR-130b overexpression induced the phosphorylation of IKK and decreased IκB expression. In contrast, miR-130b overexpression with CYLD restoration had no effect on IKK phosphorylation or IκB degradation (Figure 5D). Together, our results suggested that CYLD is a direct inhibitory target of miR-130b, the interaction of which with miR-130b potentially resulted in the activation of NF-κB signaling.

### miR-130b expression was positively correlated with NF-κB activation and inversely correlated with CYLD protein expression in bladder tumor samples

**Figure 6:**
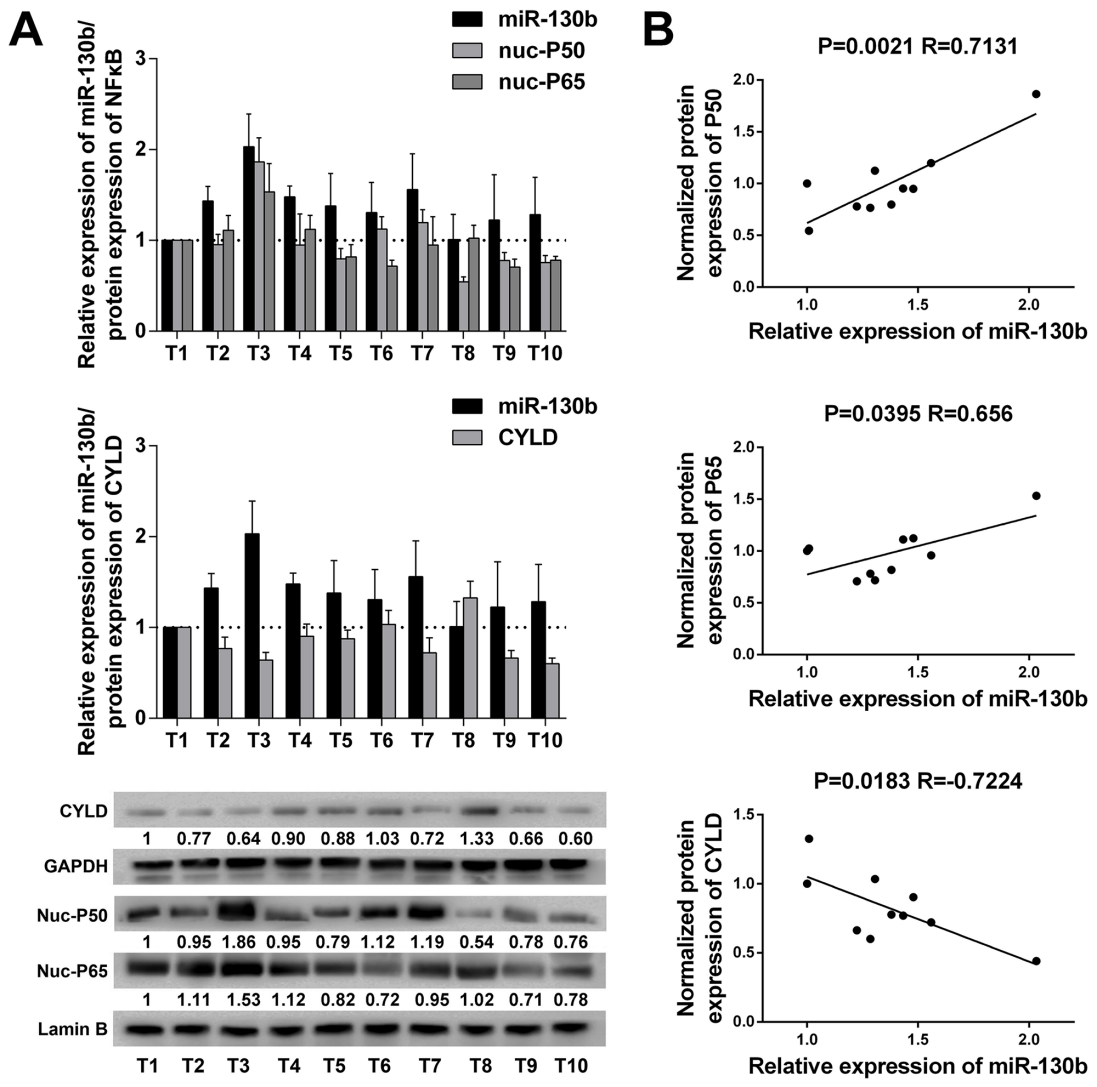
miR-130b expression was positively correlated with NF-κB activation and inversely correlated with CYLD protein expression in bladder tumor samples **A.** miR-130b expression, NF-κB subunits and CYLD protein expression in 10 bladder cancer tissue samples. The densitometric values of the bands were calculated, and relative protein expression level was normalized against the control. GAPDH and nuclear Lamin B were used as loading controls. The ratio of the first sample (CYLD/GAPDH, P50/Lamin B, P65/Lamin B) was considered as 1. **B.** The positive correlation between NF-κB and miR-130b expression and the inverse correlation between miR-130b and CYLD expression were analyzed using Spearman's correlation analysis. Each bar represents the mean ± SD of three independent experiments.

**Figure 7:**
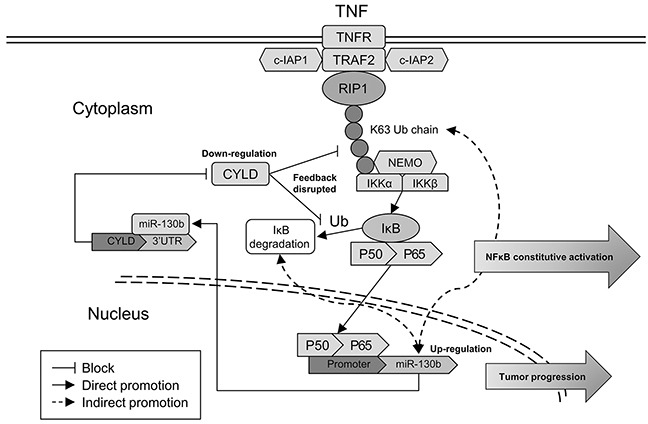
Hypothetical model illustrating the NF-κB/miR-130b/CYLD axis. Hypothetical model of NF-κB signaling activated through canonical pathway In this map, NF-κB upregulates the transcription of miR-130b, which decreases CYLD expression and disrupts the negative feedback of CYLD on NF-κB activation. This axis leads to the constitutive activation of NF-κB signaling and promotes the progression of TCC.

Finally, we examined whether the relationships between NF-κB, miR-130b, and CYLD identified in bladder cancer cells were evident *in vivo*. For this purpose, 10 bladder tumor specimens were freshly collected during radical cystectomy, and the nucleus or total proteins were extracted separately to examine the expression of NF-κB subunits or CYLD (Figure 6A). Spearman's correlation coefficient analysis revealed a significant correlation between the expression of the subunits of NF-κB in the nuclear parts and the expression of miR-130b in the samples. At the same time, the expression levels of miR-130b were negatively correlated with the protein levels of CYLD in these tumor tissues (Figure 6B). These results supported the notion that, in bladder cancer, NF-κB could sustain its activation through a feedback loop in which NF-κB induced miR-130b expression, which consequently inhibited the expression of the endogenous NF-κB blocker, CYLD. Thus, the net result of NF-κB/miR-130b/CYLD signaling was probably the persistent activation of NF-κB, which could consequently compromise the progression of bladder cancer (Figure 7).

## DISCUSSION

Accumulating evidence has shown that the dysregulation of microRNAs is closely related to the development and progression of cancers [23–25]. Several studies have also shown that the transcript factor NF-κB regulated the transcription of many microRNAs [36,37]. In this study, we found an onco-miRNA role for miR-130b in bladder cancer, and we revealed a potential scenario regarding its regulation, namely, that it sustained the activation of NF-κB signaling by decreasing the expression of the NF-κB inhibitor CYLD. To our knowledge, this report was the first to uncover the functional role and the regulatory context of miR-130b in bladder cancer.

The microRNA-130 family has been associated with the development of many cancers, probably by interfering with the processes of cell cycling, cell differentiation, EMT, and angiogenesis [27]. The involvement of miR-130b in the cell cycle and EMT has been reported previously in various cancer cell models. For example, in colorectal cancer cell lines, miR-130b directly targeted peroxisome proliferator-activated receptor γ (PPARγ), which regulates some key mediators of cell proliferation, such as P21, cyclin A, and PTEN, thus increasing cell proliferation [27]. In another study involving endometrial cancer cell lines, miR-130b suppressed DICER1 expression and led to the deregulation of miR-200, together with some other EMT-related proteins [38]. In the present study, we showed that miR-130b was involved in cell cycle control and the EMT process and, therefore, promoted cell proliferation, as well as the invasion and migration of bladder cancer cells. One mechanism underlying miR-130b regulation is that it suppressed CYLD expression and enhanced NF-κB activation, although the detailed context of this regulation remains elusive.

NF-κB plays important roles in the development and progression of cancer [39] because its constitutive activation commonly occurs in human cancers [40]. In our study, we provided evidence to demonstrate a potential novel pathway by which NF-κB sustains its constitutive activation and further promotes the progression of bladder cancer. We showed that NF-κB induced the expression of miR-130b, which in turn interrupted the inhibitory effect of CYLD on NF-κB activation. Because the activation of NF-κB signaling is dependent on the ubiquitination and degradation of endogenous inhibitors, the DUBs, such as CYLD and A20, which are able to reverse the ubiquitination and the degradation of the inhibitors, are supposed to attenuate the activation of NF-κB [7,17,41]. CYLD has been recognized as a tumor suppressor by impairing cell proliferation and inducing cell death [42–44]. In this study, we demonstrated that miR-130b suppressed CYLD expression at both the mRNA and protein levels. Finally, to support the notion that miR-130b maintains NF-κB activity by decreasing CYLD expression, we overexpressed or knocked down the expression of miR-130b in bladder cancer cells, and we confirmed the activation or suppression of NF-κB signaling, respectively. In addition, CYLD expression was restored or overexpressed by transfecting the pcDNA-CYLD plasmid, and phosphorylation of IKK/degradation of IκB was measured, with the final results further confirming our hypothesis. Thus, the present study revealed that, in bladder cancer, NF-κB can maintain its activity by establishing a feedback loop, in which NF-κB induced the expression of miR-130b, which consequently inhibited the expression of CYLD, which in turn was an endogenous inhibitor of NF-κB activation.

In summary, the present study revealed that miR-130b played an oncogenetic role in urinary bladder cancer, promoting the progression of bladder cancer by stimulating cell proliferation, invasion, and migration. In addition, it was directly regulated by NF-κB and could decrease CYLD expression, which was a crucial negative regulator of NF-κB. miR-130b appeared to play an intermediary role and promoted the persistent activation of NF-κB, thus accelerating tumorigenesis and the progression of bladder cancer. Therefore, any treatments targeting miR-130b may block this axis and inhibit NF-κB activation, and they could help us to develop a novel therapeutic approach for the management of TCC.

## MATERIALS AND METHODS

### Cell culture and transfection

Human bladder carcinoma cell lines (T24, 5637, J82, BIU-87, SW780, UM-CM-3) and an immortalized human bladder urothelial cell line (SV-HUC-1) were cultured in RPMI 1640 (HyClone, Logan, UT, USA) supplemented with 10% FBS (HyClone) and 1% penicillin-streptomycin (HyClone) at 37°C in a humidified air atmosphere with 5% CO_2_. Plasmid transfection was performed using Lipofectamine™ 3000 (Invitrogen, Carlsbad, CA, USA) according to the manufacturer's instructions. DNA plasmids were mixed with P3000™ reagents in Opti-MEM™ medium. Lipofectamine™ 3000 reagent was diluted with Opti-MEM™ medium at room temperature and was gently vortexed for 2-3 seconds. Then, DNA-P3000™ mixture was added to the diluted Lipofectamine™ 3000 reagent and incubated. After 5 minutes, the DNA-lipid complex was then added to the cells. The cell medium was replaced with complete medium after six hours, and the transfection efficiency was measured at 48 hours post-transfection.

### Tissue specimens and clinicopathological characteristics

The sixty bladder cancer tissues and ten normal bladder tissues used in our study were obtained from 60 patients who were pathologically diagnosed with bladder transitional cell carcinoma and who underwent transurethral bladder tumor resection (17 cases) or radical cystectomy (43 cases) in the Urology Department, First Hospital of China Medical University (Shenyang, China). All of the cases were classified according to the 1997 UICC TNM classification for the stage and according to OMS 2004 for the grade. Written informed consent was obtained from all of the patients prior to the study. The Institutional Research Ethics Committee approved the use of the clinical specimens for research purposes. We freshly collected 30 pairs of fresh TCC tissue specimens and matched adjacent non-tumor bladder mucosa tissue specimens (located > 3 cm from the tumor) from 30 patients and another 30 bladder cancer tissues were freshly collected from the other 30 patients. The freshly collected tissues were frozen in liquid nitrogen and stored at −80°C until used.

### TNF-α and Bay-11-7082 treatment

TNF-α was purchased from R&D systems (Minneapolis, MN, USA). After reconstitution at 100 μg/ml in sterile phosphate-buffered saline (PBS), TNF-α was stored at −80°C. It was diluted in serum-free medium to a concentration of 10 ng/ml when used and was added to the cells. Serum-free medium without TNF-α was used in the control group at the same time.

Bay-11-7082 (Selleckchem, Houston, TX, USA) in DMSO was stored at a concentration of 50 mM at −80°C. When added to the cells, Bay-11-7083 was diluted in serum-free medium to a concentration of 3 mM for T24 and 5 mM for 5637. As a control, 10 μL of DMSO was added per 1.0 ml of media.

### Plasmid and siRNA

The human pCMV4-P65 plasmid (#21966) and the relative empty vector were provided by Addgene (Cambridge, MA, USA). The human pcDNA-CYLD and relative empty vector were created by and purchased from GenePharm (Shanghai, China). The hsa-mir-130b-3p agomir, hsa-mir-130b-3p antagomir, and appropriate scrambled controls were purchased from GenePharm (Shanghai, China). The pmirGLO luciferase reporter plasmids were all created by and purchased from GenePharm (Shanghai, China).

### Western blotting

Cells were harvested in RIPA lysis buffer (Beyotime, Shenzhen, Guangdong, China) and heated for 10 min at 90°C. Protein concentrations were measured using the BCA assay. Equal amounts of protein extracts were then separated by 10% SDS-polyacrylamide gel electrophoresis (SDS-PAGE) and transferred to polyvinylidene fluoride (PVDF) membranes (Millipore, Billerica, MA, USA). The membranes were blocked with Tris-buffered saline plus Tween-20 (TBS-T; 0.1% Tween-20) with 5% (w/v) non-fat dry milk and were then incubated with primary antibodies in TBS-T at 4°C overnight. After three washes with TBS-T for 15 min each, the membranes were incubated with the appropriate HRP-labeled secondary antibodies for 1 h at 37°C. After three washes with TBS-T for 15 min each, the immunobands were visualized using the ECL reagents (Transgen Biotechnology, Beijing, China). Antibodies against P50, p65, CYLD, and IκB were purchased from Cell Signaling Technology (Danvers, MA, USA). E-cadherin, N-cadherin, phospho-IKK, IKK and lamin B1 rabbit monoclonal antibodies were purchased from Abcam (Cambridge, MA, USA). The vimentin rabbit monoclonal antibody was purchased from Santa Cruz Biotechnology (Dallas, TX, USA). The housekeeping protein GAPDH (Sigma-Aldrich, St. Louis, MO, USA) was used as an internal control for total protein measurement, and lamin B1 was used as a nucleoprotein reference. The densitometric values were calculated by AlphaEase™ FC 6.0 software (Alpha Innotech, Santa Clara, CA, USA) and the ratio of target protein to control protein was used to conduct statistical analysis.

### RNA extraction and real-time quantitative PCR

Total RNA, including micro-RNA from cultured cells and fresh surgical bladder tissues, was extracted using a miRNeasy™ Mini Kit (Qiagen, Hilden, Germany), according to the manufacturer's instructions. cDNA synthesis and quantitative real-time PCR were performed using a mercury LNA™ Universal RT microRNA PCR kit (Exiqon, Skelstedet, Vedbaek, Denmark). The has-miR-130b-3p and U6 LNA™ PCR primer sets were also purchased from Exiqon. For the detection of TNFALP3, NFKBIA, IL-8, and CYLD mRNA, total RNA was extracted by RNAiso Plus (Takara, Dalian, Liaoning, China) according to the manufacturer's protocol. cDNA was synthesized using PrimeScript™ RT Master Mix (Takara, Dalian, Liaoning, China), and quantitative real-time PCR was performed using SYBR Premix EX Taq™ (Takara, Dalian, Liaoning, China). μ-actin was used as a reference gene. The primers used to amplify the target genes are listed in Supplementary Table S1. The 2^−ΔΔCT^ method was performed to calculate the relative expression, and expression levels of negative controls were used for calibration.

### Cell cycle analysis

Cells were trypsinized and washed in ice-cold PBS and then fixed in ice-cold 75% ethanol in PBS. PI/RNase staining buffer (BD, San Diego, CA, USA) was added, and the cells were incubated at 4°C for 30 min. Cell cycle profiles were analyzed using a FACSCalibur flow cytometer (BD, San Diego, CA, USA).

### Cell proliferation assays

Cell proliferation was determined by the CCK8 assay (Dojindo, Tokyo, Japan). The cells (3 × 10^3^ cells per well) were plated in 96-well plates in 100 μL of RPMI 1640 and 10% FBS. Every 24 hours, 10 μL of CCK8 was added per well and was incubated at 37°C for 1 hour. The absorbance was measured at 450 nm using a plate reader (Model 680, Bio-Rad laboratories, Hercules, CA, USA) to determine the number of viable cells. All of the experiments were performed three times with five replications. The first measured absorbance value was defined as the baseline, and the ratios of absorbance at different time points compared with the baseline absorbance were collected to evaluate the cell proliferation rate. For the colony formation assay, the cells were plated in 24-well plates (250 cells per well) and were incubated for 10 days in complete medium. Colonies were fixed with 10% formaldehyde for 10 min and were stained with 1.0% crystal violet for 5 min. The number of colonies, defined as >50 cells/colony, was counted.

### Transwell assay

The cell invasiveness and motility were measured using transwell chambers with 8-μm pores in 24-well tissue culture plates (Corning Costar, Corning, NY, USA). Transwell chamber coated with Matrigel (BD, San Diego, CA, USA) was used to determine cell invasiveness, whereas transwell chamber without Matrigel was used to measure cell motility. After transfected with miR-130b agomir, miR-130b antagomir or their respective negative controls for 48 hours, cells were re-suspended in RPMI 1640 containing 1% FBS, and 0.2 ml cell suspension (1×10^4^/ml) was seeded into the top chamber, whereas 0.6 ml of RPMI 1640 containing 10% FBS was filled in the lower chamber, used as a chemoattractant. After 24 hours of incubation at 37°C with 5% CO_2_, the cells remained in the upper side were removed using cotton swabs, and those had migrated to the lower side were fixed and stained with 1.0% crystal violet. Images were captured (200 X), and cells were counted using ImageJ 1.48v software (National institutes of health, Bethesda, Maryland, USA).

### Xenograft tumor model and staining

BALB/c nude mice (4–6 weeks old, 14–16 g) were purchased from Beijing Vital River Experimental Animal Technology Co., Ltd. The mice were housed in barrier facilities on a 12-h light/dark cycle. The Institutional Animal Care and Use Committee of China Medical University approved all of the experimental procedures. The mice were inoculated subcutaneously with 5637 cells (5×10^6^) in the left dorsal flanks. After the volume of xenograft tumors reached approximately 3 mm^3^ (approximately 1 week after inoculation), the mice were randomly divided into two groups (n = 4/group), and the agomir of miR-130b (diluted in PBS at 2 μM) or 100 μL of negative control was injected intratumorally twice per week in either group for four weeks. Tumors were examined twice weekly; the length, width, and thickness were measured with calipers, and tumor volumes were calculated using the equation (Length × Width^2^)/2. On day 38, the animals were euthanized, and the tumors were excised, weighed, and paraffin-embedded. Serial 6.0-μm sections were cut and subjected to staining assays. The proliferation index was determined by counting the proportion of Ki67-positive cells.

### Dual luciferase reporter assay

In 24-well plates, cells (4×10^4^ cells per well) were seeded in triplicate and were cultured for 24 hours. Then, the cells were treated with TNF-α (10 ng/ml) or RNA/DNA transfection, according to the experimental purpose. Luciferase and Renilla signals were measured 48 hours after treatment using a Dual Luciferase Reporter Assay Kit (Promega, Madison, WI, USA) according to the manufacturer's protocol.

### Nuclear/cytoplasmic fractionation

A Nuclear and Cytoplasmic Protein Extraction Kit (Beyotime, Shenzhen, Guangdong, China) was used to extract the nuclear and cytoplasmic protein from culture cells and tissues, according to the manufacturer's protocol. Cells were washed with cold PBS and were resuspended in buffer containing 1 mM DTT and 1 mM PMSF, followed by incubation on ice for 15 min. Detergent was added, and the cells were vortexed for 30 s at the highest speed. The nuclei and supernatant (cytoplasm) were separated by centrifugation at 4°C. The nuclei were resuspended in buffer containing 1 mM DTT and 1 mM PMSF, were incubated on ice for 30 min, and were vortexed with interruptions. Nuclear extracts were collected by centrifugation at 14,000 × *g* for 10 min at 4°C. For nuclear protein extraction of tissues, 60 mg of frozen bladder tissues were excised and immediately suspended in buffer containing 1 mM DTT and 1 mM PMSF, were homogenized on ice, and were then incubated for 15 min. The subsequent procedure was the same as that for nuclear and cytoplasmic protein extraction.

### Chromatin immunoprecipitation assay

The chromatin immunoprecipitation (Chip) assay was performed using a SimpleChiP™ Enzymatic Chromatin IP kit (Cell Signaling Technology, Danvers, MA, USA) according to the manufacturer's protocol. Cells (4 × 10^7^) in five 150-mm culture dishes were treated with 1% formaldehyde to cross-link proteins to DNA and were collected. The chromatin was digested by micrococcal nuclease to a length of approximately 150-900 bp. The cross-linked chromatin was separately incubated with 10 μL of anti-NF-κB p50 antibody (Cell Signaling Technology), 3 μL of anti-IgG antibody (negative control, Cell Signaling Technology), or 3 μL of anti-histone H3 antibody (positive control, Cell Signaling Technology) overnight at 4°C with rotation. Protein G agarose beads were used to harvest the immunoprecipitant. After reverse cross-linking of protein/DNA complexes to free the DNA, qRT-PCR was performed using promoter-specific forward and reverse primers (Supplementary Table S2). RPL30 (provided by the kit) was used as an internal reference. Precipitated DNA was also amplified for 25 cycles and was resolved on 1% agarose gel to evaluate the amplification of target DNA.

### Statistical analysis

Each experiment was repeated three times. Data was shown as mean ± sd, all statistical analyses were carried out using SPSS 21.0 statistical software (SPSS Inc., Chicago, IL, USA). Chi-square test was used to assess the correlation between patients' clinical pathological characteristics and miR-130b expression. The cut-off point was determined by youden index to reach the highest values of sensitivity and specificity for T stage classification (>pT2). The 2-tailed Student's t-test was used to evaluate the significance of differences between two groups of data in all pertinent experiments. Spearman correlation analysis was used to compare the correlation between expression of different genes. A p-value < 0.05 was considered significant.

## SUPPLEMENTARY FIGURE AND TABLES


