# Supplementary material for: miR-130b, an onco-miRNA in bladder cancer, is directly regulated by NF-κB and sustains NF-κB activation by decreasing Cylindromatosis expression
Source: Oncotarget. 2016 Jul 6;7(30):48547–61. doi: 10.18632/oncotarget.10423 (PMC5217037; doi:10.18632/oncotarget.10423)
Supplement: Supplementary file 1 [file oncotarget-07-48547-s001.pdf]

miR-130b, an onco-miRNA in bladder cancer, is directly regulated by NF-κB and sustains NF-κB activation by decreasing Cylindromatosis expression

SUPPLEMENTARY FIGURE AND TABLES

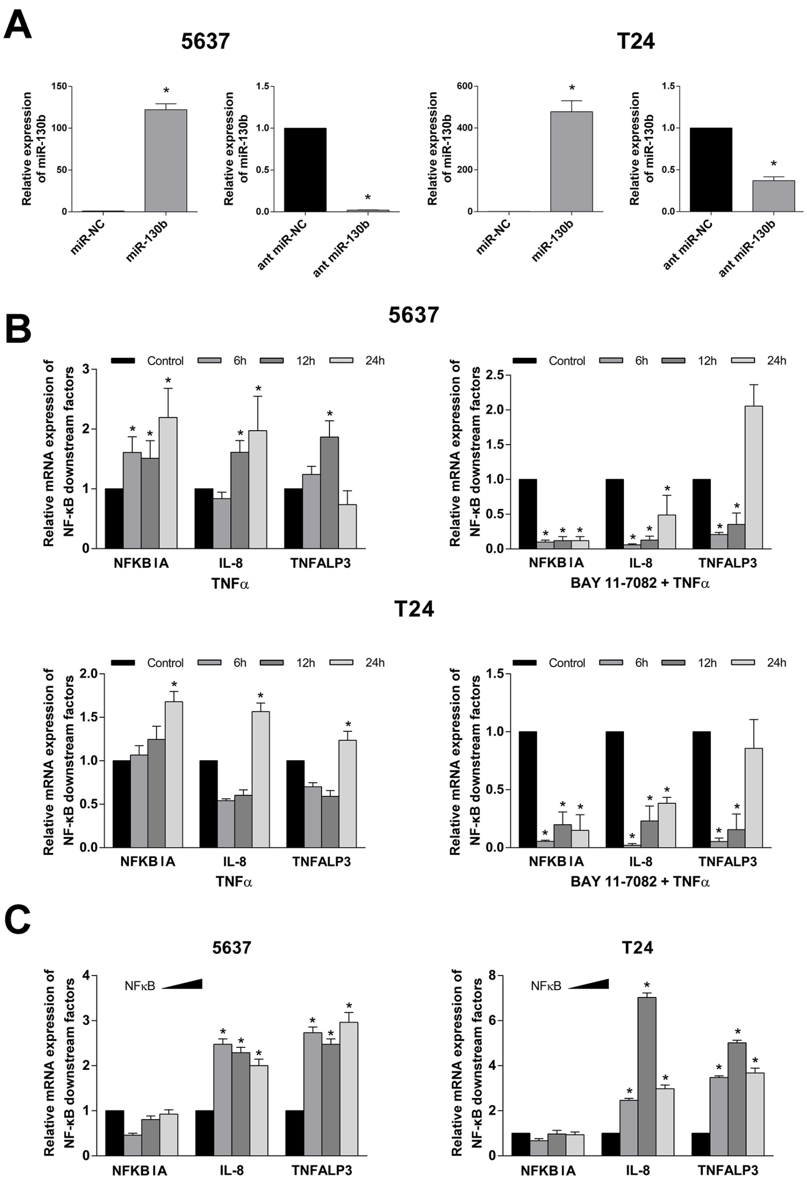

Supplementary Figure S1: A. Transfection efficiency of miR-130b agomir /antagomir. 5637 and T24 cells were transfected with miR-130b agomir or antagomir and their respective negative control. The relative miR-130b expression was analyzed by real-time PCR. And U6 served as the internal control. Each bar represents the mean ± SD of three independent experiments. $^*p < 0.05$. **B.** NF-κB downstream factors were measured after TNF-α / BAY 11-7082 treatment. Left panel: After 6, 12 and 24 hours TNF-α treatment (10ng/ml), mRNA expressions of three NF-κB downstream factors, NFKBIA, TNFALP3, IL-8 were measured by real-time PCR. β-actin was used as the control. Right panel: Cells were pretreated with BAY 11-7082 for 1 hour and then, treated with TNF-α (10ng/ml) for 6, 12 and 24 hours, mRNA expressions of three NF-κB downstream factors, NFKBIA, TNFALP3, IL-8 were measured by real-time PCR. β-actin was used as the control. Each bar represents the mean ± SD of three independent experiments. $^*p < 0.05$. **C.** NF-κB downstream factors were measured after NF-κB P65 plasmid transfection. pCMV4-P65 plasmid was transfected in increasing concentrations of 1 μg, 2.5 μg and 5 μg. After 24 hours, mRNA expressions of three NF-κB downstream factors, NFKBIA, TNFALP3, IL-8 were measured by real-time PCR. β-actin was used as the control. Each bar represents the mean ± SD of three independent experiments. $^*p < 0.05$.

Supplementary Table S1: Primers used for Real time RT-PCR

Name	5′-3′ sequence
TNFALP3 F	5′ CCTTGGAAGCACCATGTTTG 3′
TNFALP3 R	5′ TTGTGTGGTTCGAGGCACAT 3′
IL-8 F	5′ TCCTGATTTCTGCAGCTCTGT 3′
IL-8 R	5′ AATTTCTGTGTTGGCGCAGT 3′
NFKBIA F	5′ ACGAGCAGATGGTCAAGGAG 3′
NFKBIA R	5′ CTTCCATGGTCAGTGCCTTT 3′
CYLD F	5′ GGTAATCCGTTGGATCGGTCAGC 3′
CYLD R	5′ TGCAAACCTAGAGTCAGGCCTGC 3′
B-actin F	5′ ACTTAGTTGCGTTACACCCTT 3′
B-actin R	5′ GTCACCTTCACCGTTCCA 3′

F, forward; R, reverse

Supplementary Table S2: Primers of miR-130b promoters used to ChIP-qPCR analysis

Name	5′-3′ sequence	Products (bp)
miR-130b promoter-1 F	5′ CACCCATCCATGGTTGAGC 3′	183
miR-130b promoter-1 R	5′ GGAGTGGGAGGTGAGGGTTA 3′	
miR-130b promoter-2 F	5′ TTCTCCTCCACCTCAATCCG 3′	180
miR-130b promoter-2 R	5′ CCCACCACCCCCAATACA 3′	
miR-130b promoter-3 F	5′ GCCCACCACGTCCAAATA 3′	127
miR-130b promoter-3 R	5′ GCCGAGACCCTGTGGAAA 3′	

F, forward; R, reverse
